# Retrospective review: Factors impacting length of stay in Bipolar Disorder at a tertiary hospital

**DOI:** 10.4102/sajpsychiatry.v30i0.2310

**Published:** 2024-10-07

**Authors:** Nomsa C. Mkhwebane, Wendy Friedlander

**Affiliations:** 1 Department of Psychiatry, Faculty of Health Sciences, University of the Witwatersrand, Johannesburg, South Africa

**Keywords:** Bipolar Disorder, length of stay, clinical factors, socio-demographic factors, hospitalisation

## Abstract

**Background:**

Bipolar disorder (BD) is a chronic, disabling mental illness that may require recurrent hospitalisation. The length of hospital stay (LOS) for BD patients is variable, and literature suggests that this is because of clinical and socio-demographic factors.

**Aim:**

To determine the average LOS for patients admitted for BD at a hospital and its relation to clinical and socio-demographic factors.

**Setting:**

A public tertiary hospital in South Africa.

**Methods:**

Clinical and socio-demographic data were obtained from a retrospective record review of patient admissions at a hospital over 1 year. Length of hospital stay, defined as the duration between admission and discharge date, and other variables were retrieved.

**Results:**

A total of 215 patients were admitted during the study period. The mean LOS was 30 days. The mean age of the patients was 35.9 years (standard deviation [s.d.] = 12.4, range 18–72 years). There were similar numbers of males and females admitted. Significantly more patients were not married (*p* < 0.001), unemployed (*p* < 0.001), and had a history of substance use (*p* < 0.001). Employed patients were 2.5 times more likely to have a short stay than those unemployed (*p* = 0.03). There was a statistically significant association between the number of comorbidities and LOS.

**Conclusion:**

The study findings align with the literature’s results. The median length of stay was 25 days and was impacted by socio-demographic but not clinical factors.

**Contribution:**

The study provided insight into the impact of variable factors in LOS for BD patients.

## Introduction

Bipolar disorder (BD) is a chronic, disabling mood disorder characterised by episodes of severe mood disturbance associated with a profound impact on the well-being of the patient.^1^ There is a significant level of impairment associated with mental illness as a result of frequent readmissions and long-term cognitive decline. Repeated admission to the hospital is a poor prognostic indicator of severe mental illness.^2^ Consequently, patient outcomes can be improved by minimising hospital stays. A study on the Global Burden of Disease conducted in 2015 showed that BD affects approximately 44 million people worldwide.^3^ Bipolar disorder was identified as one of the top 10 ranked chronic disease list conditions (including HIV/AIDS) treated in South Africa in 2016.^4^

Hospitalisation is generally indicated in the acute manic phase mainly because outpatient treatment is unlikely to be successful because of non-adherence, disorganised behaviour and impaired functional capacity. In-patients with severe depressive symptoms and at risk to attempt suicide are additional factors to consider.^5^ Patients with BD utilise about three to four times more healthcare resources than patients without BD, mainly because of recurring hospitalisations.^6^ A study conducted in Uganda found a readmission rate of 23.8% for patients with BD within the study period. In this readmitted group, 69.0% were readmitted within the first 12 months of the discharge.^7^ The readmission rate within the first 12 months is generally lower in developed countries; Hamilton et al. found a readmission rate of 31.4%.^8^ Kessing et al. found that in Switzerland, readmission rates increased with the total number of admissions.^2,7^

Lee et al. investigated the effect of combination therapy of lithium and antipsychotics on the length of hospital stay (LOS).^9^ Their study found that about one-third of the admitted patients were on a combination regimen, which reduced the LOS by 2.8 days compared to monotherapy. The concern with the combination regimen is tolerability, especially in older patients with comorbid conditions.^9^ The literature recommends that combination regimens should be used when faster response is needed, in severe manic episodes, and when there is a history of partial response to monotherapy previously.^10^ The use of first-generation antipsychotics is commonly associated with extrapyramidal side effects (EPSE).^11^ The development of EPSE will prolong the LOS as additional treatment and further close monitoring are mandatory once adverse effects develop.^12^ It would then be recommended that first-generation antipsychotics are avoided in high-risk patients, and if used, active monitoring for any adverse effects is required.

Electroconvulsive treatment (ECT) is the oldest and most efficient therapy that can be used for a manic episode.^1^ In acute mania and treatment-resistant BD, a response rate ranging between 80% and 90% has been reported with the use of ECT.^13^ Despite the well-documented positive response, ECT is still considered the last resort. A study conducted in Sweden found that ECT was better than psychotropics.^14^ The general recommendation from this research was that ECT be considered early after admission. In the absence of the delay in initiating the treatment, the LOS was reduced by up to 14.7 days.^15^ In contrast, a Nigerian study found that ECT was the only treatment-related variable significantly associated with prolonged LOS.^16^ However, further analysis suggests that patients who require ECT generally have severe illness and will, therefore, require prolonged admission.^11^

Patients with treatment-resistant BD are usually challenging to manage because of their poor response to known effective regimens. Fornaro et al. investigated predictors of LOS in patients with an established history of pharmacologically resistant BD in Europe. In this study, it was found that female gender, current use of second-generation antipsychotics and being on an antidepressant were associated with prolonged admission.^17^ Interestingly, the mean LOS for this group of patients was 30.45 days, similar to the mean LOS for patients with treatment-sensitive BD in developing settings. A Nigerian study found a mean LOS of 25 days.^16^ The use of substances is very common in patients with BD and could impact the adherence to treatment and overall outcome of patients with chronic mental illness.^18^ In one study, lifetime alcohol dependence was associated with extended length of stay.^18^ A Malawian study found that substance use was associated with short LOS.^11^ The strong desire to access the substances is likely the primary motivation. It is this anticipated freedom to have access to substances outside the hospital environment that could result in shortened hospitalisation in those known with a history of substance use on admission.^11^

Martin-Carraso et al. found that a high number of previous episodes and the presence of depressive symptoms are associated with prolonged LOS.^19^ Ukpung et al. also found that previous multiple inpatient admissions are associated with prolonged LOS.^16^ Medical comorbidity can have an impact on the length of stay. Douzenis et al. investigated the effect of active comorbidity on the length of stay in patients admitted with BD and schizophrenia.^20^ In their review, active medical comorbidity was described as a medical condition that required medical consultation during hospitalisation.^20^ It was found that patients admitted with BD who required two reviews of their underlying medical condition during their hospitalisation, had prolonged median LOS of 5.5 days more.^20^

Literature suggests that socio-demographic factors have a significant impact on the LOS. Ismail et al. looked at age as a factor in LOS by comparing geriatrics and adult patients with mood disorders. It was found that geriatrics had more extended hospitalisation.^21^ Similarly, male patients above the age of 50 years were found to have prolonged hospitalisation in another study.^1^ The employed and married patients were found to have a shorter LOS.^16^ This may be because the capacity to maintain a relationship and be productive in the workplace indicates a less severe illness or that marriage and employment are protective.^22^ Additionally, there may be a more significant motivation for early recovery and discharge to return to work and family.

Ismail et al. investigated LOS in geriatrics and found that living alone negatively impacted LOS. A safe discharge for someone living alone requires reasonable symptom control and a high level of functional independence compared to someone with a close support structure.^21^ The literature reviewed highlights that the average LOS for patients admitted with BD varies globally, and further research to determine the contributing factors would be helpful, particularly in the local context.

## Aims and objectives

The study aimed to determine the LOS in patients with BD and determine which (if any) clinical and socio-demographic factors impact this.

The objectives of this study are:

- To determine the average length of stay of patients hospitalised with BD over the study period from 01 January 2022 to 31 December 2022.
- To determine whether any demographic and clinical factors affect the length of hospitalisation.

## Research methods and design

### Study design

This study is an institution-based, retrospective record review of patients managed for BD at a hospital in South Africa.

### Study setting

The tertiary hospital provides psychiatric services to a large population from around the area. In addition, it receives numerous patients referred from district-level hospitals, which lack the resources and capacity to manage severely ill or complex patients. The period reviewed was from 01 January 2022 to 31 December 2022.

### Study population

The study population included all patients admitted to the hospital during the study period diagnosed with BD. The most recent admission was included in patients with more than one admission in the year. The inclusion criteria were in-patients diagnosed with BD as per the Diagnostic and Statistical Manual of Mental Disorders, Fifth Edition (DSM-V), patients aged 18 years and older, and patients admitted to the hospital for acute management of BD (mania or depression). Patients who were still hospitalised by the end date of the study period, pregnant women (to mitigate the effect of pregnancy on LOS), and patients who absconded or died before discharge were excluded from the analysis.

### Statistical analyses

The length of stay in the hospital was described as the total number of days between the admission and discharge dates. The data were captured in Microsoft Excel^TM^. Statistical analyses were conducted using R software (version 3.4.2;^2^). Tests are two-tailed probability values, and statistical significance was accepted when *α* ≤ 0.05. Continuous variables and categorical data were reported accordingly. The association between length of stay and multiple variables was analysed using Pearson’s chi-squared or Fisher’s tests. Further analysis included binary post-hoc tests and pairwise Fisher’s tests. Odds ratios are reported for significant Fisher’s *p* values. The continuous variables were assessed for departure from normality using the Shapiro-Wilk test and subsequently analysed using a Mann–Whitney *U* test.

### Ethical considerations

Ethical clearance to conduct this study was obtained from the University of the Witwatersrand, Human Research Ethics Committee (No. M230443). Written permission was obtained from the hospital management. Privacy and confidentiality of the participants were maintained by ensuring that no identifying information was on the data collection form; instead, the researcher allocated the participants a unique number associated with their clinical records, and the linked file was kept separately. Signed patient informed consent was not required for the record review.

## Results

This retrospective study included 215 patients. Sample sizes in the analyses might vary because of missing data for some variables. The mean age of the patients was 35.95 years (s.d. = 12.45, range: 18–72 years). Table 1 summarises the socio-demographic and clinical profiles of the study participants. The number of females did not differ significantly from that of males (Table 1). In contrast, significantly more patients were not married than married, and significantly more patients were not employed than employed (Table 1). For the polarity of the episodes, significantly more patients had manic episodes than depressed and mixed episodes (binary post hoc), and the majority of patients had no comorbidities compared to those with 1, 2 and 3 or more comorbidities. There was a significantly higher number of patients who used substances than those without a history of substance use. Most patients had more than two previous admissions compared to those with 0–2 admissions (binary post hoc). In the category of patients on psychotropic medications on discharge, significantly more patients were on two psychotropics compared to those on 0, 1, 3 and 4 psychotropics (binary post hoc), and only one patient was on no psychotropic. Significantly more patients did not have EPSE than those who did. Finally, none of the patients were treated with ECT (see Table 1).

**TABLE 1:** The socio-demographic and clinical profile of patients with bipolar disorder at a hospital.

Variable	Count	Per cent	Statistics
**Sex**			*χ*^2^ = 0.907, *df* = 1, *p* = 0.341
Female	115	53.0	-
Male	101	47.0	-
**Married**			***χ*^2^ = 124.52, *df* = 1, *p* < 0.001**
No	190	88.0	-
Yes	26	12.0	-
**Employment**			***χ*^2^ = 112.67, *df* = 1, *p* < 0.001**
No	186	86.0	-
Yes	30	14.0	-
**Manic or depressive condition**			***χ*^2^ = 374.19, *df* = 2, *p* < 0.001**
Depressive	7	3.0	-
Manic	206	95.0	-
Mixed	3	1.0	-
**Number comorbidities**			***χ*^2^ = 130.78, *df* = 3, *p* < 0.001**
0	124	57.0	-
1	49	23.0	-
2	25	12.0	-
≥ 3	18	8.0	-
**Substance use**			***χ*^2^ = 15.11, *df* = 1, *p* < 0.001**
No	79	37.0	-
Yes	136	63.0	-
**Number of previous admissions**			***χ*^2^ = 134.00, *df* = 3, *p* < 0.001**
0	27	13.0	-
1	35	16.0	-
2	26	12.0	-
> 2	127	59.0	-
**Number of psychotropic drugs on discharge**			***χ*^2^ = 308.95, *df* = 3, *p* < 0.001**
0	1	0.5	-
1	14	6.0	-
2	141	65.0	-
3	50	23.0	-
4	10	5.0	-
**EPSE**			***χ*^2^ = 114.65, *df* = 1, *p* < 0.001**
No	186	86.0	-
Yes	29	13.0	-

Note: Statistics = Pearson’s chi-squared tests; Bold numbers reflects statistical significance and *p*-value < 0.05.

EPSE, Extrapyramidal side effects.

**FIGURE 1:**
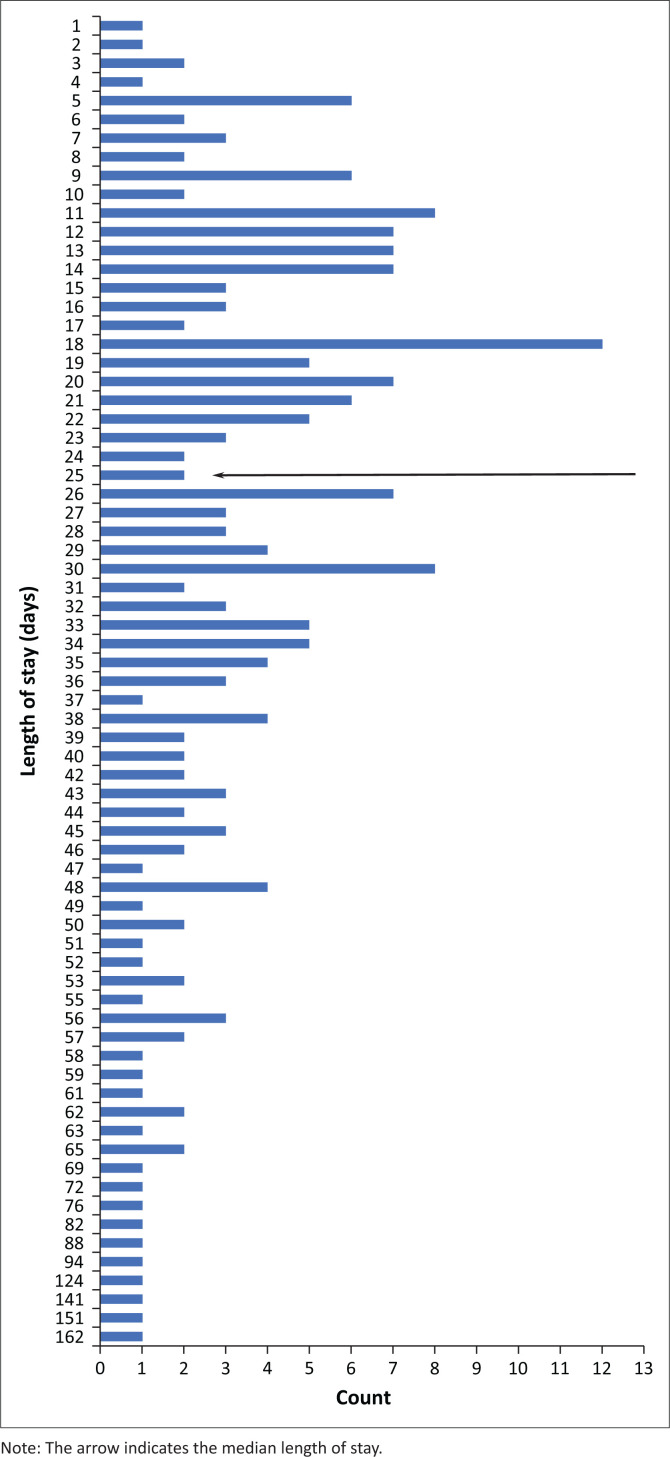
Frequency distribution of the length of stay in patients with bipolar disorder at a hospital.

The median length of stay was 25 days (1st interquartile range [IQR] = 14 days, 3rd IQR = 38 days). The mean length of stay was 30 days (s.d. = 23.89, IQR 1–162 days). Based on the median score, the length of stay of patients was divided into short-stay patients (< 25 days) and long-stay patients (≥ 25 days). Figure 1 shows a frequency distribution of the length of stay in patients in this study. Most patients spent 18 days in hospital.

The age of the patients was not a significant predictor of the length of stay (W = 5377.00, *p* = 0.682). Most other socio-demographic and clinical variables were not significant predictors of the length of stay (Table 2). However, employment and the number of comorbidities were significant predictors of the length of stay (Table 2). Employed patients were 2.5 times more likely to have short stays than those not employed (odds ratio [OR]). Patients with one comorbidity were 2.7 and 3.9 times more likely to have short stays than those with no and two comorbidities, respectively. Similarly, those with three or more comorbidities were 1.7 times more likely to have a short stay than those without.

**TABLE 2:** The association between socio-demographic and clinical variables and length of stay in patients with bipolar disorder at a hospital.

Variable	Long stay		Short stay		Statistics
	Count	Per cent	Count	Per cent	
**Sex**					*p* = 0.343
Female	60	52.0	55	48.0	-
Male	46	45.0	55	54.0	-
**Married**					*p* = 0.292
No	96	50.0	94	49.0	
Yes	10	38.0	16	61.0	
**Employment**					***p* = 0.031**
No	96	52.0	90	48.0	-
Yes	9	30.0	21	70.0	-
**Manic or depressive condition**					*χ*^2^ = 1.57, *df* = 2, *p* = 0.547
Depressive	2	29.0	5	71.0	-
Manic	102	49.0	104	50.0	-
Mixed	2	67.0	1	33.0	-
**Number of comorbidities**					***χ*^2^ = 11.31, *df* = 2, *p* = 0.010**
0	68	55.0	56	45.0	-
1	15	31.0	34	69.0	-
2	16	64.0	9	36.0	-
≥ 3	7	39.0	11	62.0	-
**Substance use**					*p* = 0.888
No	38	48.0	41	52.0	
Yes	67	49.0	69	51.7	
**Number of previous admissions**					*χ*^2^ = 2.50, *df* = 3, *p* = 0.476
0	17	63.0	10	37.0	-
1	16	46.0	19	54.0	-
2	12	46.0	14	54.0	-
> 2	60	47.0	67	53.0	-
**Number of psychotropic drugs on discharge**					*χ*^2^ = 6.09, *df* = 3, *p* = 0.193
0	0	0.0	1	100.0	-
1	6	43.0	8	57.0	-
2	63	45.0	78	55.0	-
3	31	62.0	19	38.0	-
4	6	60.0	4	40.0	-
**EPSE**					*p* = 0.322
No	89	48.8	97	52.0	-
Yes	17	59.0	12	41.0	-

EPSE, extrapyramidal side effects.

Note: Statistics = Fisher’s tests (*p*-values only) or Pearson’s chi-square tests; Bold numbers reflects statistical significance and *p*-value < 0.05.

## Discussion

This study aimed to determine the LOS and factors associated with prolonged length of stay in patients admitted to the hospital with BD. The median length of stay was 25 days (1st IQR = 14 days, 3rd IQR = 38 days). The mean length of stay was 30 days (s.d. = 23.89, IQR 1–162 days). This median LOS was comparable to a study conducted by Wang et al. which found a median LOS of 28 days in 4451 patients.^23^ Another study of 170 BD patients found an average LOS of 37 days, significantly higher than the current study.^24^ Compared with other African studies, a Nigerian study reported a mean LOS of 25 to 23 days, while an Ethiopian study reported a mean LOS of 22 days.^12,16^ A Malawian study found the length of stay in their facility to be 22.08 ± 27.20 days.^11^ These comparisons must be made considering that the above African studies included all psychiatric conditions and did not focus exclusively on BD.

The variability of LOS across different countries may be because of resource availability, which impacts the quality of psychiatric care. The lack of access to other medications may potentially delay the clinical response and recovery of patients admitted with BD. It has been reported that some institutions have limited access to atypical antipsychotics and other effective drugs that are used commonly.^11,12^

Concerning the socio-demographic variables, there were no significant differences in the numbers of males and females, but the majority of the patients were single, unemployed and used substances. The similarities in the presentation of males and females are congruent with a previous study on BD that found a 1:1 presentation of males and females.^25^ Although some studies have found differences in the presentation of males and females according to types of BD, this was not the case in this review.^26,27^ This can partly be explained by the overwhelming majority of patients presenting with a manic episode. It appears that patients are more likely to be brought to the hospital when manic, probably because of the level of disruption experienced by those around them.

Gender was also not a statistically significant predictor of prolonged stay in the BD patients in this sample. These findings are congruent with a study conducted in Malawi.^11^ Interestingly, other studies have shown that the male gender predicts prolonged LOS in BD patients.^1^ The association between gender and length of stay for BD is disputable because confounding factors weaken and better explain this association.^28^ Comorbid alcohol and substance use have been reported to affect length of stay, and their prevalence is different for males compared to females. Similarly, comorbid personality disorder, which is more common in females, has been found to prolong hospitalisation.^28^ Single-site study and non-representative biased study samples are some of the factors contributing to different study outcomes.^28^ Treating hospital practices, which are often influenced by the availability of psychiatric beds and medication, accessibility to health care providers and increased costs associated with hospitalisation, have been found to affect LOS, specifically shortened length of stay.^29^

In this study, age was also not a significant predictor of prolonged LOS. This finding concurs with the Malawian study.^11^ But it differs from the study conducted by Bodicherla et al., who found that LOS increased with age.^1^ In this sample, marital status was not a significant predictor of LOS. This was congruent with the findings of the study by Barnett et al. and contradicted the findings of the Nigerian research that found that marriage was associated with shorter LOS.^11,30^ The low proportion of married participants in the sample may impact this.

Employment was a significant predictor of LOS; employed patients had shorter stays than those not employed. These findings were congruent with those of a study conducted in China, which found that unemployment was associated with prolonged LOS.^24^ This finding has several potential reasons: Firstly, employment indicates high psychosocial functioning in patients with BD.^31^ This is a good prognostic indicator for recovery. Secondly, the symptoms of employed individuals may be detected earlier (because of impairment in functioning and alterations in behaviour), allowing timeous treatment seeking, which can minimise the length of stay. However, this was not found in the Nigerian study, where employment was not associated with LOS.^30^ The study conducted by Adegunloye did not focus specifically on patients admitted with BD, and more than half (i.e., 55.0%) of their patients were diagnosed with schizophrenia.^30^ Therefore, the results of their study may not be directly generalisable to this study population.

Regarding the clinical characteristics, the polarity of the disorder (manic/depressive) was not a significant predictor of LOS. The majority of the patients in this sample presented with mania, thus making the sample of patients with depressive symptoms inadequate to generate statistical significance. The findings on episode polarity are congruent with the study conducted by Shi et al., which found that polarity was not a significant predictor of LOS.^24^ A study done by Fornaro et al. echoed similar findings.^17^

On the other hand, comorbidities were a significant predictor of LOS. The study by Shi et al. found that comorbid anxiety disorders did not increase LOS in their sample.^24^ This difference may be attributed to the fact that the current study did not assess specific comorbidities but rather the number of comorbidities. Conditions such as anxiety may be managed without hospitalisation in resource-constrained environments. Studies of medical comorbidities among psychiatric patients also show that comorbidities do increase LOS.^20^ This could be because of the condition’s impact or the associated polypharmacy.

This study found that the number of patients with comorbid substance abuse was significantly higher in the group with prolonged length of stay compared to the short stay group. However, neither variable was an independent predictor of LOS in this study. These findings again contradict previous studies that show comorbid substance abuse to be an independent predictor of LOS. Patients with comorbid substance use might be admitted with symptoms secondary to misuse rather than BD symptoms. Therefore, this might lead to a shorter hospital stay as the symptoms generally resolve quickly.^28^ Ragana et al. reported in their study that patients with a positive history of alcohol or other substance use had significantly shortened lengths of hospital stay.^28^ In this current review, the type of substance used was not analysed, and a large percentage of the participants were found to have used substances. Further research with a study design powered to eliminate confounders would better evaluate the predictive power of substance use on the length of stay for BD.

Similar to the findings on LOS, the findings on independent predictors of LOS vary from study to study, necessitating a need for context-specific research. In their study, Harman et al. concluded that about 11% of the variation in the length of stay for psychiatric disorders could be attributed to the treatment practices at the facility where patients are being treated.^32^

## Strengths and limitations

The quality of the data collected is dependent on how comprehensively the information was captured initially. Patients are usually admitted with an acute manic episode or unstable condition, which may prevent the accurate collection of socio-demographic data and past medical history. The study includes patients from only one unit, which will affect generalisability. Each clinician in an institution likely has a similar way of managing BD. This is borne out by the absence of ECT use in any of the patients. The diagnosis cannot be corroborated since it is a retrospective review where there is always a risk of information bias. Literature on the length of stay for hospitalised patients with BD has investigated extensively the impact of socio-demographic factors. It is well documented that the results may not be fully applicable to other populations because of the retrospective nature of the studies, small sample size and other study limitations. In the same way, this review did not identify specific comorbidities that are known to individually contribute to prolonged length of stay because of the nature of the disorder. Additionally, the substance use variable was not fully described in terms of the nature of use and type of substance.

Although this study provides South African data on LOS in BD patients for the first time, there were limitations. This study could not determine whether or not some of the observed associations were related to institutional practices. Future research using primary data such as interviews would improve the robustness of these findings.

## Conclusion

This study’s findings are comparable with those from other studies in low- and middle-income countries and developed countries. Notwithstanding the limitations, the study provides data regarding the socio-demographic and clinical factors that impact the LOS in BD patients admitted to a hospital. This has predictive value for optimising care and minimising admission duration in patients with BD. This, in turn, may result in better patient outcomes and cost savings for the institution. Multicentre studies are recommended to expand the cohort size, which can increase the statistical power and enhance the generalisability of the findings.
